# Gomisin N Inhibits Melanogenesis through Regulating the PI3K/Akt and MAPK/ERK Signaling Pathways in Melanocytes

**DOI:** 10.3390/ijms18020471

**Published:** 2017-02-22

**Authors:** Jae Kyoung Chae, Lalita Subedi, Minsun Jeong, Yong Un Park, Chul Young Kim, Hakwon Kim, Sun Yeou Kim

**Affiliations:** 1 College of Pharmacy, Gachon University, #191, Hambakmoero, Yeonsu-gu, Incheon 21936, Korea; jae1990@nate.com (J.K.C.); subedilali@gmail.com (L.S.); minsun.jeong@gmail.com (M.J.); kong_park@naver.com (Y.U.P.); 2 College of Pharmacy, Institute of Pharmaceutical Science and Technology, Hanyang University, Ansan 15588, Korea; chulykim@hanyang.ac.kr; 3 Department of Applied Chemistry and Institute of Natural Sciences, Kyung Hee University, Global Campus, #1732 Deogyeong-daero, Giheung-gu, Yongin, Gyenggi-do 17104, Korea; hwkim@khu.ac.kr; 4 Gachon Medical Research Institute, Gil Medical Center, Inchon 21565, Korea; 5 Gachon Institute of Pharmaceutical Science, Gachon University; #191 Hambakmoe-ro, Yeonsu-gu, Incheon 21565, Korea

**Keywords:** *Schisandra chinensis*, Gomisin N, lignan, melanogenesis, skin whitening

## Abstract

Gomisin N, one of the lignan compounds found in *Schisandra chinensis* has been shown to possess anti-oxidative, anti-tumorigenic, and anti-inflammatory activities in various studies. Here we report, for the first time, the anti-melenogenic efficacy of Gomisin N in mammalian cells as well as in zebrafish embryos. Gomisin N significantly reduced the melanin content without cellular toxicity. Although it was not capable of modulating the catalytic activity of mushroom tyrosinase in vitro, Gomisin N downregulated the expression levels of key proteins that function in melanogenesis. Gomisin N downregulated melanocortin 1 receptor (MC1R), adenylyl cyclase 2, microphthalmia-associated transcription factor (MITF), tyrosinase, tyrosinase-related protein-1 (TRP-1), and tyrosinase-related protein-2 (TRP-2). In addition, Gomisin N-treated Melan-A cells exhibited increased p-Akt and p-ERK levels, which implies that the activation of the PI3K/Akt and MAPK/ERK pathways may function to inhibit melanogenesis. We also validated that Gomisin N reduced melanin production by repressing the expression of MITF, tyrosinase, TRP-1, and TRP-2 in mouse and human cells as well as in developing zebrafish embryos. Collectively, we conclude that Gomisin N inhibits melanin synthesis by repressing the expression of MITF and melanogenic enzymes, probably through modulating the PI3K/Akt and MAPK/ERK pathways.

## 1. Introduction

Melanin is a pigment found in most animal organs including skin, hair, eyes, inner ear, bones, heart, and brain [1,2]. Melanogenesis is a complex process where multiple signaling pathways are involved. The melanocortin 1 receptor (MC1R) is a key regulator in melanogenesis, signaling through its ligands such as melanocyte-stimulating hormone (MSH) and adrenocorticotropic hormone (ACTH) [3]. Skin melanin is biosynthesized by melanocytes in the epidermis and then transferred to keratinocytes, where they play important roles in skin protection by absorbing UV radiation from sunlight and scavenging reactive free radicals [4,5]. Melanin synthesis and transfer in the skin and hair follicles is regulated by the availability of its precursors [6]. l-tyrosine and l-dihydroxyphenylalanine (l-DOPA), major substrates of melanogenic enzymes, also function as hormone-like regulators in melanogenesis [7]. On the other hand, the overproduction of melanin results in undesirable skin concerns such as freckles and melasma [8,9]. Melanogenesis can affect the behavior of normal and malignant melanocytes by modulating the elastic properties of the cells [10]. Although the receptors for serotonin and melatonin expressed in cutaneous cells play a key role in maintaining cellular homeostasis, the excessive production of melanin via uncontrolled hormonal changes may cause pathological conditions in the skin [11].

Thus, there have been extensive efforts to elucidate molecular mechanisms that control melanogenesis as the primary step for treating hyperpigmentary skin disorders. In addition, various types of skin whitening agents that inhibit melanin synthesis have been identified from plant and non-plant extracts and commercially used in cosmeceuticals [12,13]. The most widely used whitening agents include hydroquinone, mequinol, arbutin, Kojic acid, ascorbic acid, and retinoic acid [12,14]. However, there are various limitations of their use in treating acute or chronic symptoms of hyperpigmentation in humans. For example, hydroquinone, although it has long been used for depigmentation since the 1960s, can cause skin irritation and contact dermatitis [15,16]. It also leads to DNA damage by increasing the production of reactive oxygen species and the development of exogenous ochronosis in mammalian cells [17,18]. Other well-known tyrosinase inhibitors such as Kojic acid and ascorbic acid not only have poor skin penetration, stability, and whitening efficacy but also can cause cytotoxicity, dermatitis, and erythema from long-term use [19,20]. In this regard, there is an increasing need to develop safer and more effective whitening agents for treating human skin hyperpigmentation. Natural herbs used in traditional medicine may provide alternative sources for identifying novel whitening agents that control key steps in melanogenesis with less or no side effects [21].

**Figure 1:**
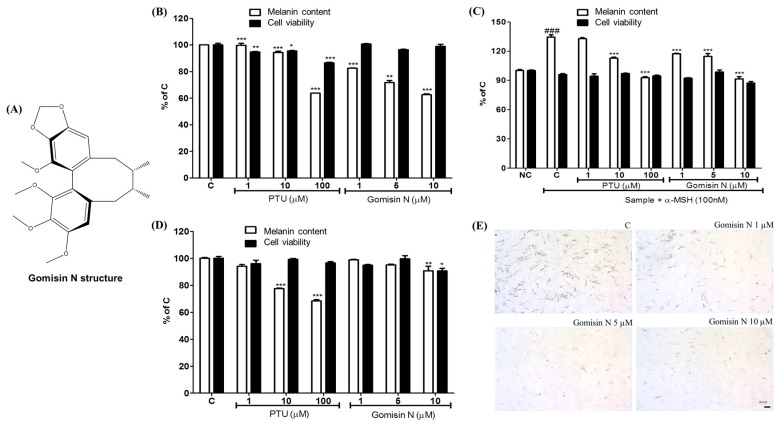
Gomisin N structure and its effects on melanin production and cell viability. (**A**) Chemical structure of Gomisin N. Melanin contents and cell viability after 72 h of Gomisin N (1, 5, 10 µM) treatment in Melan-A cells (**B**), B16F10 cells (**C**), and in MNT1 cells (**D**). PTU (1, 10, 100 µM) was used as a positive control. (**E**) Melanin contents in NHEM were measured by l-DOPA staining. Scale bar is 100 µm. The results are presented as a percentage of the vehicle-treated control and as the mean ± SD of three independent experiments. * *p* < 0.05, ** *p* < 0.01, and *** *p* < 0.001 versus the vehicle-treated control (C). ### *p* < 0.001 versus the untreated control (NC). Scale bar = 100 µm.

*Schisandra chinensis*, also known as northern fine-flavor berry, is naturally found in northeastern China, far-east Russia, Japan, and Korea [22]. This plant has long been used for the treatment of night blindness, skin burn, aseptic inflammation, and liver diseases in traditional oriental medicine [23,24]. *S. chinensis* fruit extract and its lignan compounds have been shown to possess various pharmacological effects in murine cell lines. For example, schisandrin A and C have anti-oxidative effects, while schisandrin B has anti-fibrotic, anti-inflammatory, anti-oxidant, and anti-apoptotic activities [25]. Another lignan compound Gomisin N (Figure 1A) has been reported to suppress oxidative stress-induced apoptosis by inhibiting the release of cytochrome C from mitochondria to the cytoplasm, the cleavage of caspase 3 and PARP, and Ca^2+^-induced mitochondrial permeability transition in H9c2 rat cardiomyocytes [7,8]. Interestingly, several studies using mouse models and human skin cell lines have revealed the therapeutic potential of *S. chinensis* for treating skin disorders. Lee et al. reported that the methanol extract of *S. chinensis* fruit alleviated contact dermatitis symptoms by reducing the production of pro-inflammatory cytokines such as TNF-α and IFN-γ when topically applied [8,24]. Kang et al. showed that *Schisandra* fructus water extract inhibited IκB activation, thereby suppressing the production of TNF-α, IL-6, and GM-CSF in the human mast cell line HMC-1 [26]. These findings led us to postulate that *S. chinensis* lignans might affect skin cell functions more broadly. In this study, we sought to investigate the putative roles of Gomisin N, one of the major lignan compounds in *S. chinensis*, in regulating melanogenesis, thereby evaluating its potential use as a cosmeceutical agent. Here, we report that Gomisin N inhibits melanin biosynthesis without cellular toxicity in human and mouse cells, as well as in zebrafish embryos.

## 2. Results

### 2.1. Effects of Gomisin N on Melanin Formation and Cell Viability

To verify the effects of Gomisin N on melanogenesis, we treated mouse melanocytes with different concentrations of Gomisin N for 72 h, and then assessed changes in melanin contents. Gomisin N treatment reduced the melanin content both in the normal melanocyte cell line Melan-A and in B16F10 melanoma cells in a dose-dependent manner without cellular toxicity (Figure 1B,C). We observed that Gomisin N inhibited α-MSH-induced melanin production in B16F10 cells, which was comparable with the results obtained by PTU treatment [27] (Figure 1C). To evaluate whether the reduction of melanin upon Gomisin N treatment is due to decreased activity of tyrosinase, we performed l-DOPA staining assay in normal human epidermal melanocyte (NHEM) cells. As shown in Figure 1E, NHEM cells treated with Gomisin N exhibited decreased levels of l-DOPA compared with untreated cells. However, the inhibitory effect of Gomisin N on melanin production was not significant in human melanoma MNT-1 cells (Figure 1D).

### 2.2. Effects of Gomisin N on Tyrosinase Activity

**Figure 2:**
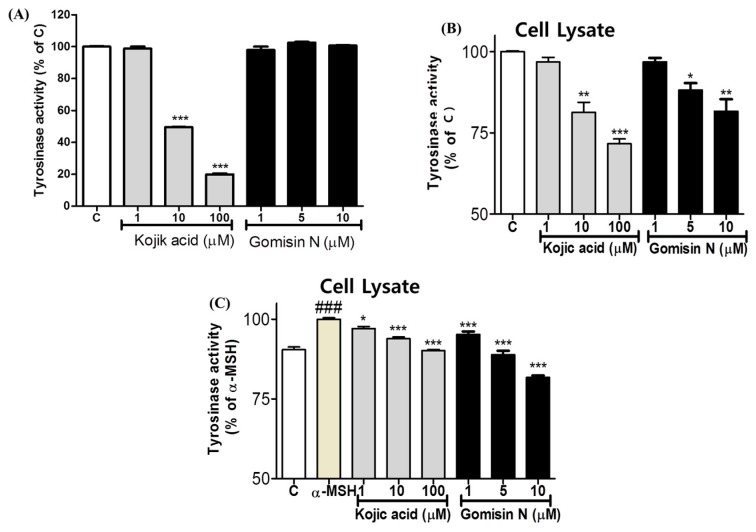
Effects of Gomisin N on mushroom tyrosinase activity. The effect of Gomisin N treatment (1, 5, 10 µM) on mushroom tyrosinase activity was measured using Gomisin N compound (**A**) and cell lysates from Gomisin N-treated B16 cells without (**B**) or with (**C**) α-MSH stimulus. Kojic acid was used as a positive control. All data are presented as the mean ± SEM of three independent experiments. * *p* < 0.05, ** *p* < 0.01, *** *p* < 0.001, and ### *p* < 0.001, versus the untreated control.

**Figure 3:**
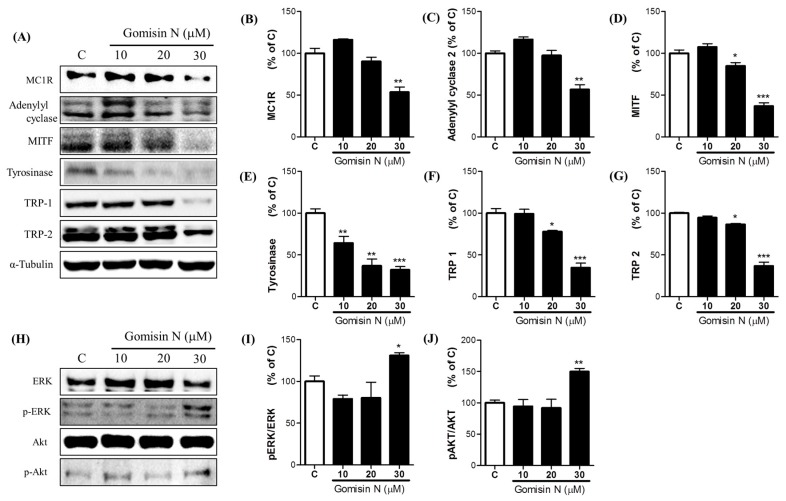
Effects of Gomisin N on the MC1R-mediated melanogenic pathways in Melan-A cells. Melan-A cells were treated with 1, 5, or 10 µM of Gomisin N for 72 h, and then subjected to Western blot analysis. Western blot (**A**) and densitometric analysis (**B**–**G**) for MC1R, adenylyl cyclase 2, MITF, tyrosinase, TRP-1, and TRP-2; Western blot (**H**) and densitometric analysis (**I**,**J**) for ERK, p-ERK, Akt, and p-Akt. α-Tubulin was used as a loading control. All data are presented as the mean ± SEM of three independent experiments. * *p* < 0.05, ** *p* < 0.01, and *** *p* < 0.001 versus the untreated control (C).

To examine whether Gomisin N inhibits tyrosinase activity in vitro, we used mushroom tyrosinase and B16 melanoma cell lysates. Kojic acid, a well-known tyrosinase inhibitor, was used as a positive control. When treated to mushroom tyrosinase, whereas Kojic acid significantly reduced the enzymatic activity, Gomisin N did not induce any change in the conversion of l-DOPA to dopachrome (Figure 2A). However, when treated to B16 melanoma cells, Gomisin N resulted in a decrease in tyrosinase activity of the cell lysate in a dose-dependent manner (Figure 2B). Moreover, when co-treated with α-MSH in B16 cells, Gomisin N reversed the increase in dopachrome formation stimulated by α-MSH (Figure 3C). Notably, Gomisin N appeared to be more effective than Kojic acid to inhibit the cellular tyrosinase activity of B16 cells upon α-MSH stimulus. These findings imply that the inhibitory effect of Gomisin N on melanin production observed in mouse and human cells (Figure 1) is not owing to its function to directly inhibiting the catalytic activity of tyrosinase. However, it is still possible that Gomisin N regulates the expression of tyrosinase or other proteins that play key roles in melanogenesis.

### 2.3. Effects of Gomisin N on the Inactivation of the MC1R Signaling Pathway

We sought to elucidate the underlying mechanism responsible for the inhibitory effect of Gomisin N on melanin production. We reasoned that Gomisin N might regulate signaling proteins that are involved in melanogenesis and thereby inhibit melanin synthesis. Melanocortin 1 receptor (MC1R) is a melanocytic G protein-coupled receptor that functions as a key regulator in melanin synthesis. The activation of MC1R by its ligand α-MSH or adrenocorticotropic hormone (ACTH) leads to an increase in adenylyl cyclase, which in turn upregulates intracellular cAMP levels [2,3]. Consequently, the transcriptional level of MITF is increased via the Protein kinase-C (PKA)/responsive element binding protein (CREB) pathway [2,28]. To evaluate the regulatory effect of Gomisin N on the MC1R signaling pathway, we checked the expression levels of MC1R and its downstream signaling molecules after treating Melan-A cells with Gomisin N. We observed that Gomisin N significantly downregulated the protein levels of both MC1R and adenylyl cyclase 2 in a dose-dependent manner (Figure 3A–C). As expected, Gomisin N-treated cells also exhibited decreased protein levels of MITF and its known targets tyrosinase, TRP-1, and TRP-2 (Figure 3A,D–G). These findings suggest that Gomisin N inhibits melanin-producing enzymes by inactivating MITF via the MC1R signaling pathway.

### 2.4. Effects of Gomisin N on the Phosphorylation of Akt and ERK1/2 in Melan-A Cells

The PI3K/Akt and MAPK/ERK pathways are known to be involved in melanogenesis by transcriptionally or post-transcriptionally regulating MITF [29,30]. To evaluate whether Gomisin N affects these signaling pathways, we assessed the phosphorylation status of Akt and ERK1/2 by Western blot analysis. As shown in Figure 3H–J, high-dose treatment (30 µM) of Gomisin N significantly enhanced the phosphorylation of both Akt and ERK. These data indicate that the inhibitory effect of Gomisin N on melanogenesis is likely to be associated with the P13K/Akt and MAPK/ERK pathways.

### 2.5. Gomisin N Inhibited Melanogenesis in Zebrafish Embryos

**Figure 4:**
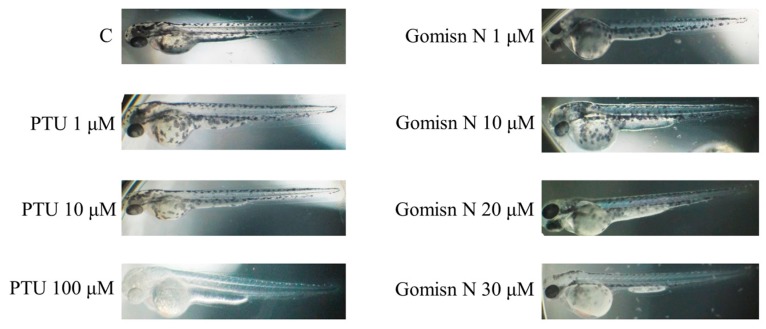
Effects of Gomisin N on inhibition of melanogenesis in zebrafish. Melanin formation in developing zebrafish embryos was observed under a stereomicroscope after treatment with 10, 20, or 30 µM of Gomisin N for 72 h. PTU (1, 10, 100 µM) was treated as a positive control.

**Figure 5:**
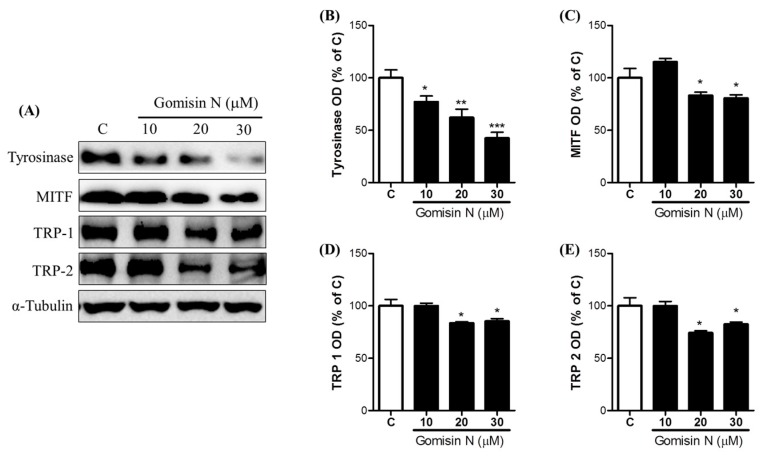
Effects of Gomisin N on the melanogenic pathways in zebrafish. Protein levels of tyrosinase (**A**,**B**), MITF (**A**,**C**), TRP-1 (**A**,**D**), and TRP-2 (**A**,**E**) were measured by Western blot analysis in zebrafish embryos after treatment with 10, 20, or 30 µM of Gomisin N. α-Tubulin was used as a loading control. Densitometric data are presented as the mean ± SEM of three independent experiments. * *p* < 0.05, ** *p* < 0.01, and *** *p* < 0.001 versus the untreated control (C).

We next aimed to examine whether Gomisin N is effective in inhibiting melanogenesis in vivo. To this end, we treated zebrafish embryos with Gomisin N for 72 h at concentrations of 1, 10, 20, and 30 µM, and then measured expression levels of melanogenic proteins. We observed that Gomisin N treatment inhibited melanin formation in developing zebrafish embryos. Gomisin N-treated embryos showed a reduction in melanin contents in a concentration-dependent manner compared with the untreated control (Figure 4). We also found that the protein levels of tyrosinase, MITF, TRP-1, and TRP-2 were decreased by Gomisin N treatment (Figure 5). These results demonstrate that Gomisin N inhibits melanogenesis in vivo by regulating the transcription factor MITF and its targets tyrosinase, TRP-1, and TRP-2.

### 2.6. Gomisin N reversed Rapamycin-induced Melanogenesis in Human MNT-1

#### Melanoma Cells

**Figure 6:**
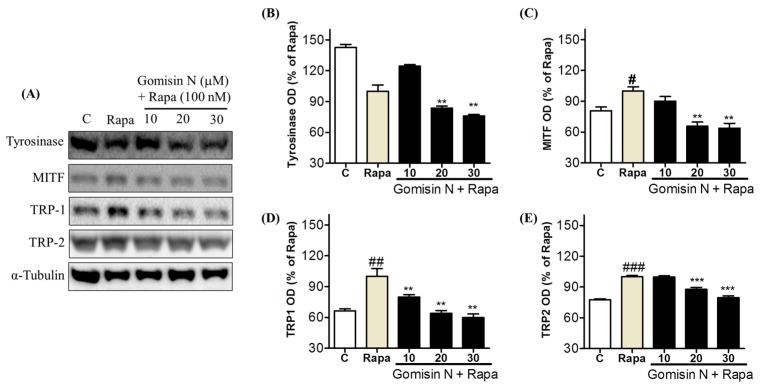
Effects of Gomisin N on the melanogenic pathways in rapamycin-stimulated MNT-1 cells. Protein levels of tyrosinase (**A**,**B**), MITF (**A**,**C**), TRP-1 (**A**,**D**), and TRP-2 (**A**,**E**) were measured Western blot analysis in MNT-1 cells after co-treatment with 10, 20, or 30 µM of Gomisin N and 100 nM of rapamycin for 72 h, α-Tubulin was used as a loading control. Densitometric data are presented as the mean ± SEM of three independent experiments. ** *p* < 0.01, and *** *p* < 0.001 versus the rapamicin-treated control (Rapa). # *p* < 0.05, ## *p* < 0.01, and ### *p* < 0.001 versus the untreated control (C).

Although Gomisin N significantly inhibited melanogenesis in Melan-A and B16 cells as well as in zebrafish embryos, we did not observe its effect on human MNT-1 melanoma cells. We expected that the effect of Gomisin N might be detectable in MNT-1 cells under the condition where melanogenesis is upregulated by an appropriate stimulus. Rapamycin has been shown to induce melanogenesis by increasing tyrosinase activity and protein levels of MITF, tyrosinase, TRP-1, and TRP-2 [31], partially through the activation of autophagy [32]. We monitored the levels of tyrosinase, MITF, TRP-1, and TRP-2 in MNT-1 cells by Western blot analysis, after co-treatment with Gomisin N and rapamycin. Rapamycin treatment significantly induced MITF, TRP-1, and TRP-2 levels but had no effect on tyrosinase levels (Figure 6). However, Gomisin N treatment significantly reversed the effects of rapamycin on MITF, TRP-1, and TRP-2 in a concentration-dependent manner. The reverse effect of Gomisin N against rapamycin was more promising at the concentration of 20 and 30 μM than 10 μM. These results suggest that Gomisin N inhibits melanogenesis in the human MNT-1 melanoma cells by regulating the transcription factor MITF and its targets TRP-1 and TRP-2.

## 3. Discussion

The main function of melanin is to protect skin cells against UV radiation [33,34,35]. Hyperpigmentation, the result of an overproduction of melanin in the skin, causes unwanted cosmetic concerns, and is associated with dermatitis and skin cancer. Several reports have suggested melanogenesis as an important target for treating metastatic melanoma [36,37]. Thus, there is an increasing need to develop anti-melanogenic agents that regulate melanogenesis without cellular toxicity [38]. There are several pathways involved in skin melanogenesis [39,40]. Upon ligand binding, MC1R enhances the activity of adenylyl cyclase, which subsequently increases intracellular levels of cAMP [41,42]. The cAMP-dependent activation of the PKA/CREB pathway has widely been reported to upregulate transcriptional levels of MITF, thereby enhancing melanin synthesis [43]. MITF functions as a master regulator of the three major melanogenic enzymes tyrosinase, TRP-1, and TRP-2 in vertebrates [3,21,44]. These enzymes are transmembrane proteins located in the melanosomal membrane of melanocytes. Tyrosinase regulates the rate-limiting step in melanogenesis by converting l-tyrosinase to l-DOPA [23]. TRP-1 and TRP-2 also play important roles in melanin synthesis, although their functions are not fully understood.

In this study, Gomisin N, a lignan compound of *S. chinensis* showed depigmenting activity without cellular toxicity. Gomisin N inhibited melanin synthesis in cultured mammalian cell lines as well as in zebrafish embryos. Gomisin N seemed to be more effective than the positive control PTU in inhibiting melanin production in Melan-A cells (Figure 1B). Gomisin N reduced the melanin content in a concentration-dependent manner. Compared with the untreated control group, 10 µM of Gomisin N reduced the melanin content by about 40%, without cellular toxicity. The anti-melanogenic activity of 10-µM Gomisin N was comparable with that of 100-µM PTU in Melan-A cells. Similarly, Gomisin N appeared to be more potent than PTU in α-MSH-activated B16F10 cells, where the effects of 5- and 10-µM Gomisin N were comparable with those of 10- and 100-µM PTU, respectively (Figure 1C). NHEM cells treated with Gomisin N exhibited reduced levels of l-DOPA, which suggests that Gomisin N inhibits tyrosinase activity in cultured cells (Figure 1E). These findings led us to further investigate the underlying mechanism by which Gomisin N inhibits melanogenesis.

We examined whether Gomisin N directly modulates the catalytic activity of tyrosinase in vitro. Unlike Kojic acid, Gomisin N did not show inhibitory effects on mushroom tyrosinase activity (Figure 2A). However, the cellular tyrosinase activity in B16 melanoma cell lysates was significantly downregulated by Gomisin N both with and without α-MSH treatment (Figure 2B,C). Inhibition of cellular tyrosinase activity of Gomisin N upon α-MSH stimulus was found to be more significant than that of the positive control Kojic acid (Figure 2C).

We postulated that the anti-melanogenic function of Gomisin N might occur through transcriptional or post-transcriptional regulation of tyrosinase and tyrosinase-related proteins (TRPs). To validate this, we measured expression levels of signaling molecules in the MC1R pathway, a major determinant for the quantity and quality of melanin production in melanocytes. Expectedly, we observed that Gomisin N reduced the levels of MC1R and adenylyl cyclases 2 in Melan-A cells (Figure 3A–C). Furthermore, Gomisin N downregulated the expression of MITF and its target proteins including tyrosinase, TRP-1, and TRP-2 (Figure 3A,D–G). These results suggest that the reduced levels of melanin contents upon Gomisin N treatment result from the deactivation of the MC1R pathway.

On the other hand, the PI3K/Akt and MAPK/ERK pathways can phosphorylate MITF, and thereby can post-transcriptionally modulate its activity [45]. However, the overall effect of the activation of the PI3K/Akt and MAPK/ERK pathways in melanogenesis is controversial. Both the PI3K/Akt and MAPK/ERK pathways are constitutively activated in human melanomas due to accumulated mutations [46]. C_2_-ceramide-mediated depigmentation in Mel-Ab cells is known to occur through a reduction in p-Akt levels [47]. There are several natural compounds that activate melanogenesis by upregulating p-ERK levels in B16 melanoma cells [28]. In contrast, there is also evidence that elevated p-ERK and p-Akt levels inhibit melanin synthesis [28,48]. The complexity in regulation of melanogenesis can be partially explained by the fact that phosphorylation enhances transcriptional activity of MITF, but simultaneously induces ubiquition-proteosome-dependent degradation of MITF [26,49,50,51]. Our data showed that both p-Akt and p-ERK levels were upregulated in Gomisin N-treated Melan-A cells (Figure 3H–J). This implies that the PI3K/Akt and MAPK/ERK pathways may contribute to the inhibition of melanin production.

We further validated the anti-melanogenic activity of Gomisin N in the zebrafish in vivo model. Gomisin N-treated zebrafish embryos showed a significant reduction in melanin pigmentation (Figure 4). In addition, Gomisin N markedly decreased the levels of tyrosinase, MITF, TRP-1, and TRP-2 in developing zebrafish embryos. These findings collectively suggest that Gomisin N induces depigmentation by downregulating the expression of MITF and melanogenic enzymes in vivo. The anti-melanogenic activity of Gomisin N was further confirmed in human melanoma MNT-1 cells stimulated by rapamycin. Although Gomisin N resulted in only small changes in melanin content in MNT-1 cells (Figure 1D), it was effective to reverse rapamycin-induced upregulation of MITF, TRP-1, and TRP-2 in a concentration-dependent manner (Figure 6A,C–E). Taken together, the regulatory effect of Gomisin N on MITF and melanogenic enzymes was reproducibly found in mouse and human cells as well as in zebrafish embryos.

**Figure 7:**
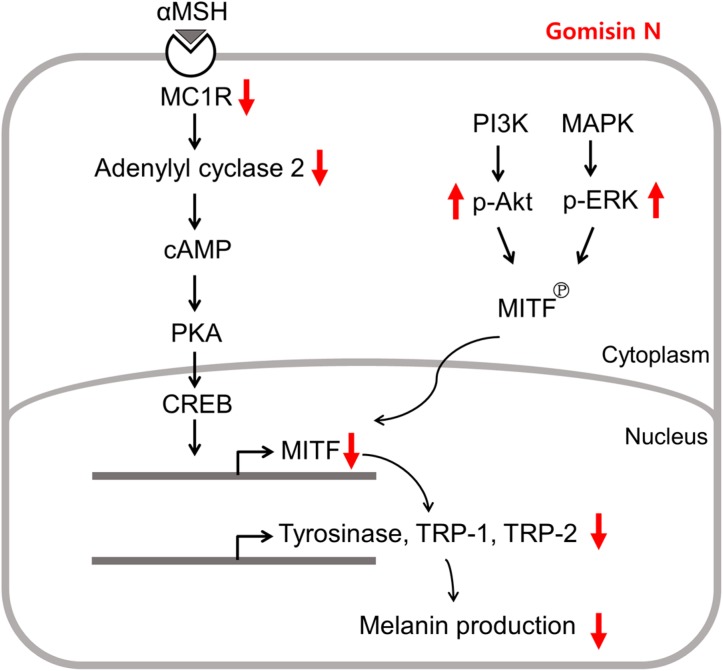
Schematic description of changes in melanogenesis upon Gomisin N treatment. Red arrow define the activity of Gomisin N.

To summarize, this work suggests that Gomisin N may have a high potential as a novel skin-whitening agent. Gomisin N appears to inhibit melanogenesis by repressing the expression of MITF via the MC1R pathway, instead of directly modulating the catalytic activity of tyrosinase and TRPs. Although detailed mechanisms remain to be elucidated, Gomisin N-induced depigmentation is likely to be associated with the activation of the PI3K/Akt and MAPK/ERK pathways (Figure 7).

## 4. Materials and Methods

### 4.1. Materials

RPMI1640 was purchased from Gibco-BRL (Gaithersburg, MD, USA). Dulbecco’s modified Eagle’s medium (DMEM), fetal bovine serum (FBS), and penicillin-streptomycin (PS) were purchased from Hyclone (Carlsbad, CA, USA). Melanocyte growth medium was purchased from PromoCell (Heidelberg, Germany). Phenylmethylsulfonyl fluoride (PMSF), 12-*O*-tetradecanoylphorbol-13-acetate (TPA), Kojic acid, 1-phenyl-2-thiourea (PTU), mushroom tyrosinase, 3,4-dihydroxy-l-phenylalanin (l-DOPA), α-MSH, dimethyl sulfoxide (DMSO), and paraformaldehyde were purchased from Sigma Chemical Co. (St. Louis, MO, USA). Gomisin N compound was provided by Chul Young Kim (Hanyang University, Ansan, Korea). Rapamycin was purchased from Sigma-Aldrich (St. Louis, MO, USA).

### 4.2. Cell Culture

The mouse melanoma cell line B16F10 was provided from the Korean Cell Line Bank (Seoul, Korea). Murine melanocyte Melan-A cells [52] were a generous gift from Dr. Byeong Gon Lee (the Skin Research Institute, Amore Pacific Co., Yongin-si, Korea). Human MNT-1 melanoma cells were generously provided by Aeyeong Lee (Collage of Medicine in Dongguk University, Goyang-si, Korea). Primary normal human epidermal melanocytes (NHEM) were purchased from PromoCell (Heidelberg, Germany). Melan-A cells were grown in RPMI 1640 medium (Gibco, Carlsbad, CA, USA) supplemented with 10% FBS, 1% PS, and 200 nM TPA. DMEM supplemented with 10% FBS and 1% PS was used to maintain Melan-A cells and NHEM cells. All cells were incubated at 37 °C in a 5% CO_2_ incubator.

### 4.3. Measurement of Melanin Contents

Melan-A cells were seeded in a 24-well plate (1 × 10^5^ cells/well), treated with Gomisin N, and then incubated for 72 h. After 72 h, the melanin content was measured as previously described [53]. Briefly, after removing the culture media, the cells were washed three times with PBS. Thereafter, sodium hydroxide solution (1 mL, 1 N) was added to each well to dissolve melanin. The absorbance at 405 nm was measured using a microplate reader. This assay was repeated with B16F10 cells (2 × 10^4^ cells/well) and MNT-1 cells following the same method.

### 4.4. Western Blot Analysis

Melan-A cells were seeded in 100 mm dishes (1 × 10^6^ cells/dish) and treated with 1, 5, or 10 µM Gomisin N for three days at 37 °C. Cells were washed with PBS and then harvested using a scraper. Detached cells were put in 1 ml of PBS and centrifuged at 7500 rpm for 5 min. After removing the upper solution, cell pellets were lysed with lysis buffer (50 mM Tris-HCl, pH 8.0, 0.1% SDS, 150 mM NaCl, 1% NP-40, 0.02% sodium azide, 0.5% sodium deoxycholate, 100 µg/mL PMSF, 1 g/mL approtinin) for 24 h at 4 °C. Total proteins were extracted using an ultracentrifuge at 12,000 rpm for 30 min at 4 °C. The protein content was measured using Bradford assay. Proteins (30 µg) were separated using a 10% sodium dodecyl sulfate polyacrylamide gel electrophoresis (SDS-PAGE) gel and transferred to a nitrocellulose membrane. The membrane was blocked for 1 h with 5% skim milk in Tris-buffered saline with Tween-20 (TBST), and then incubated for 12 h at 4 °C with primary antibodies targeting α-tubulin (Santa Cruz, CA, USA), MITF (Cell Signaling, Danvers, MA, USA), tyrosinase (Cell Signaling), ERK (Cell Signaling), phospho-ERK (Cell Signaling), AKT (Cell Signaling), phospho-AKT (Cell Signaling), MC1R (Santa Cruz), adenylyl cyclases 2 (Santa Cruz), TRP-1 (Santa Cruz), and TRP-2 (Santa Cruz). After removing primary antibodies, membranes were washed three times with TBST and incubated with secondary antibodies (rabbit anti-goat IgG-HRP; mouse anti-rabbit HRP, Santa Cruz) for 1 h. The membranes were treated with enhanced chemiluminescence reagent using the ChemiDoc XRS+ imaging system (Bio-Rad, Hercules, CA, USA). Densitometric analysis of the bands was performed using Image Master^TM^ 2D Elite software (version 3.1, GE Healthcare, Chicago, IL, USA).

### 4.5. Tyrosinase Activity Assay

To estimate the inhibitory effect of Gomisin N on mushroom tyrosinase activity, tyrosinase was incubated with 1, 5, or 10 µM Gomisin N or the positive control Kojic acid. Each sample was dissolved in methanol. l-DOPA (8.3 mM) and mushroom tyrosinase (125 U) were diluted in 80 mM phosphate buffer (pH 6.8). 40 µL of each sample and 120 µL of L-DOPA were mixed in a 96-well plate, followed by the addition of 40 µL of diluted mushroom tyrosinase. The plates were then incubated for 15 min, and absorbance was measured at 490 nm using a microplate reader.

Tyrosinase activity in B16 melanoma cell lysates was measured with or without α-MSH treatment, as previously described by Ohguchi et al. [54], with slight modifications. Cell lysate was prepared as described above in the Western Blot analysis part. Total proteins in the supernatant were measured by Bradford assay using Bovine Serum Albumin as a standard [55]. An equal amount of proteins was diluted and used for the tyrosinase activity assay.

### 4.6. l-DOPA Staining in NHEM Cells

NHEM cells were seeded in a 24-well plate and incubated for 72 h with Gomisin N. Cells were fixed with 4% paraformaldehyde for 40 min, followed by treatment with 0.1% triton X-100 for 2 min. l-DOPA (0.1%) was added to each well, followed by incubation for 2 h. After removing the solution, the cells were washed twice with PBS. Images were photographed by microscope.

### 4.7. Zebrafish Experiments

Zebrafish embryos were obtained from the Zebrafish Resource Bank (Daegu, Korea). Embryos were treated with Gomisin N for 72 h. The depigmenting effect of Gomisin N on zebrafish embryos was observed under the stereomicroscope. For Western blot analysis, Gomisin N-treated embryos were lysed using lysis buffer, from which total proteins were prepared as mentioned above.

## 5. Conclusions

Our result supports the view that Gomisin N has high potential for use as a functional food and skin-whitening agent. Gomisin N is one of the major lignan compounds in *S. chinensis*. In fact, *S. chinensis* is an herbal medicine used for the cure of many human diseases. However, further epidemiological studies are necessary to prove the safety of Gomisin N on the skin. Consequently, in vivo study and clinical trials will be able to more clearly demonstrate the effectiveness of Gomisin N. In conclusion, this study suggests that Gomisin N may be a potential hypo-pigmentary agent and natural skin-whitening candidate for the cosmetic industry.
